# Supplementary material for: Characterisation of the OTU domain deubiquitinase complement of Toxoplasma gondii
Source: Life Sci Alliance. 2023 Mar 23;6(6):e202201710. doi: 10.26508/lsa.202201710 (PMC10038098; doi:10.26508/lsa.202201710)
Supplement: Supplementary file 1 [file LSA-2022-01710_Supplemental_Data_1.pdf]

# TgOTUD1

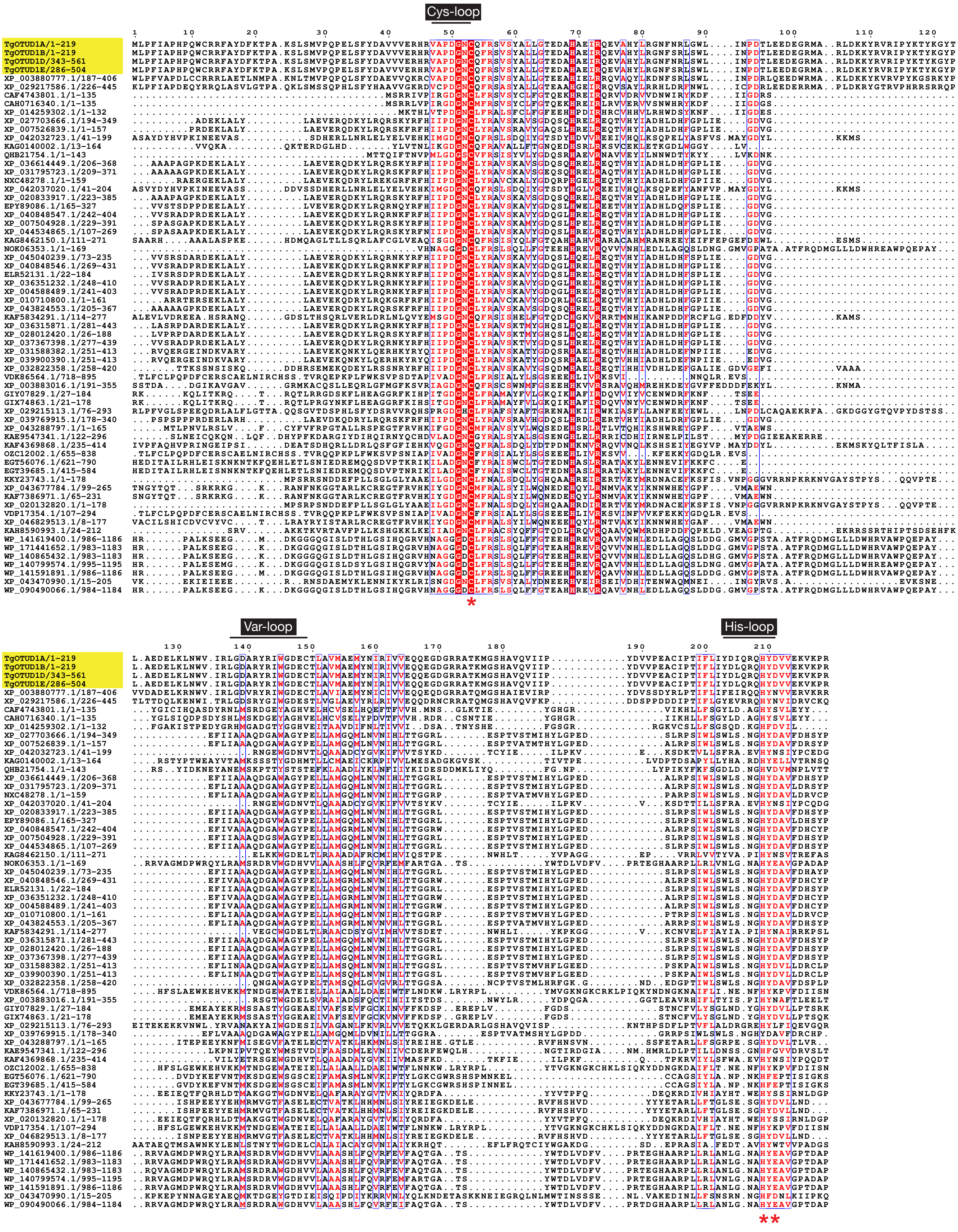

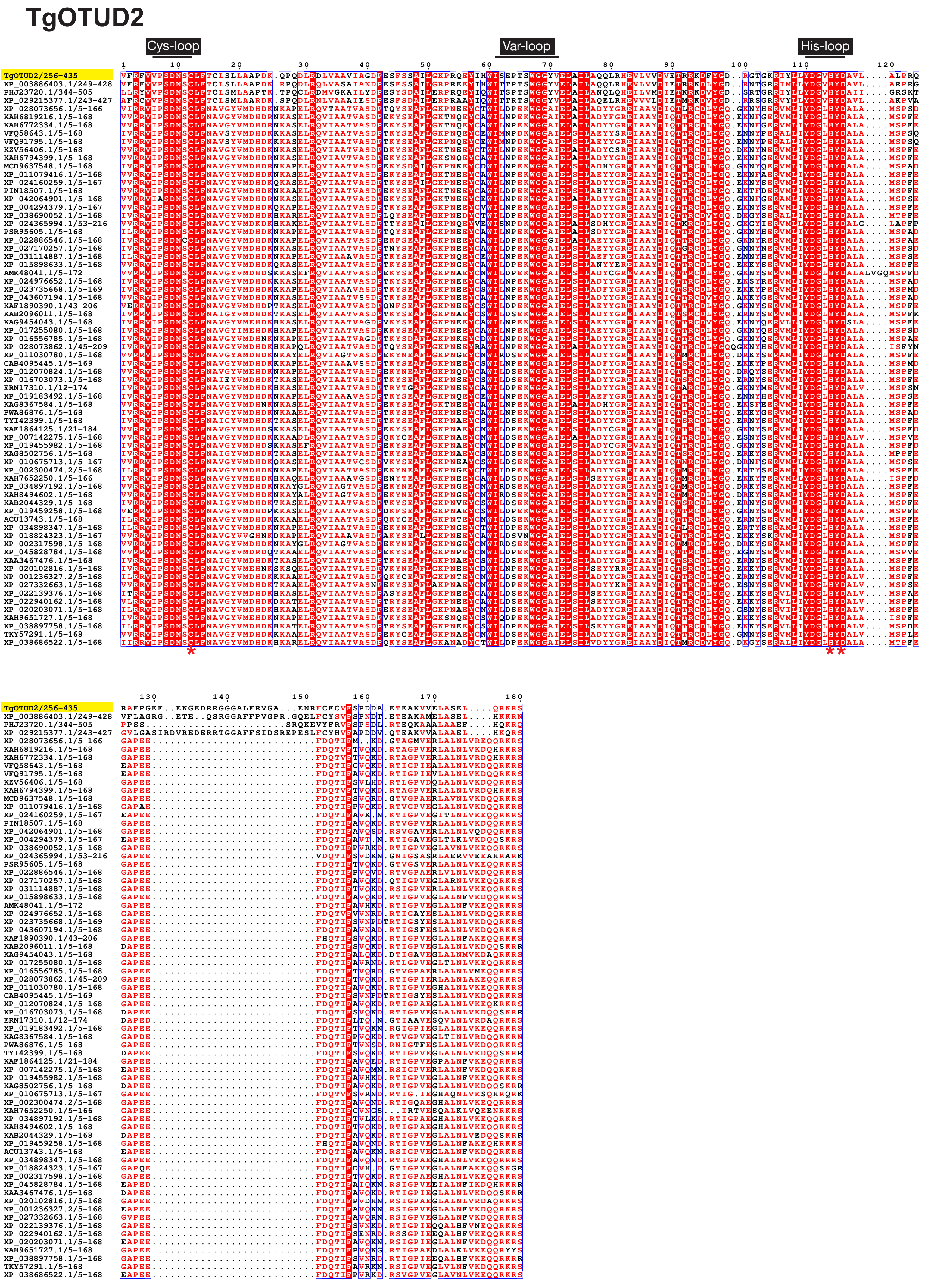
TgOTUD2
Cys-loop
Var-loop
His-loop

# TgOTUD3A

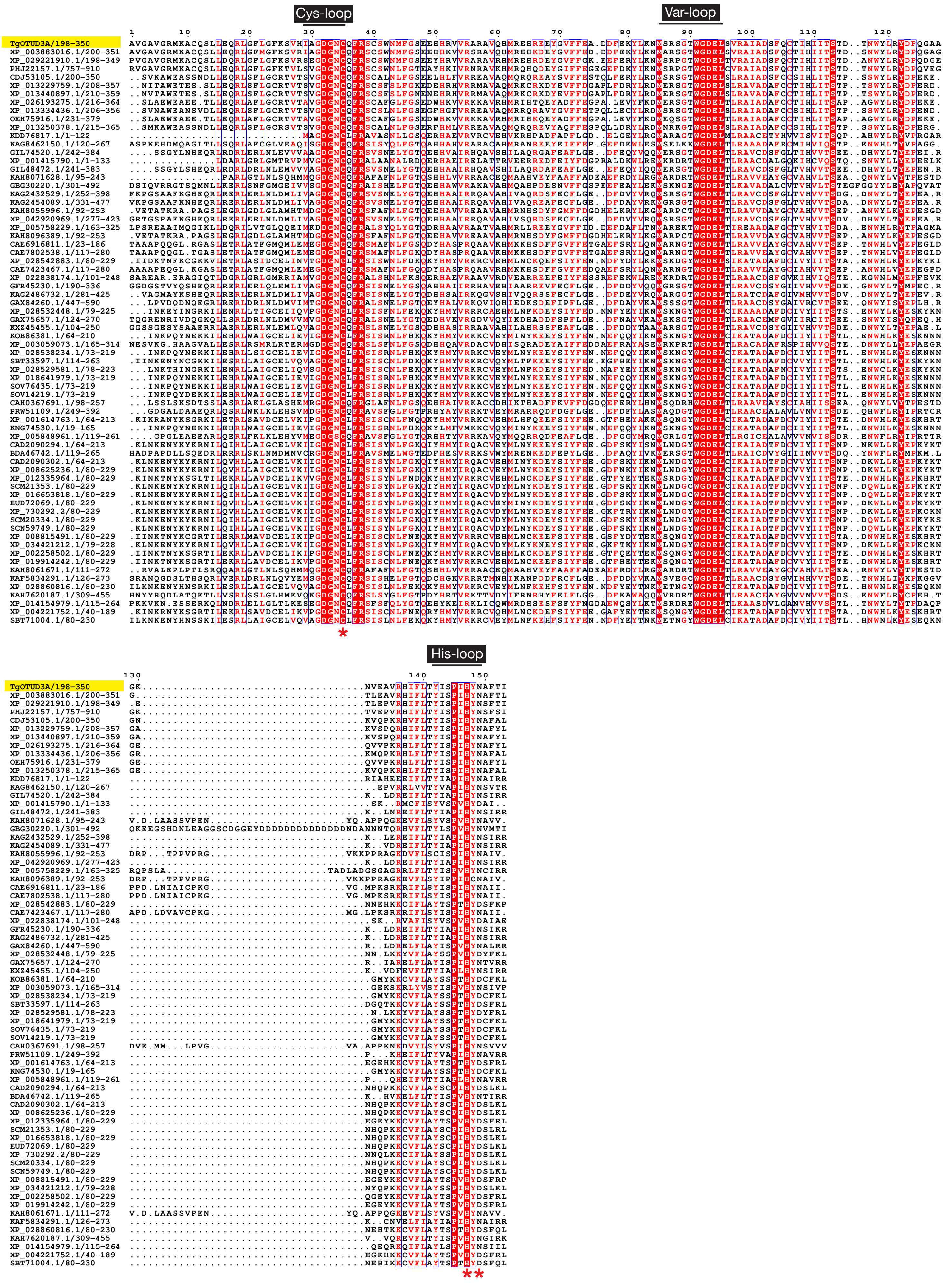

# TgOTUD3B

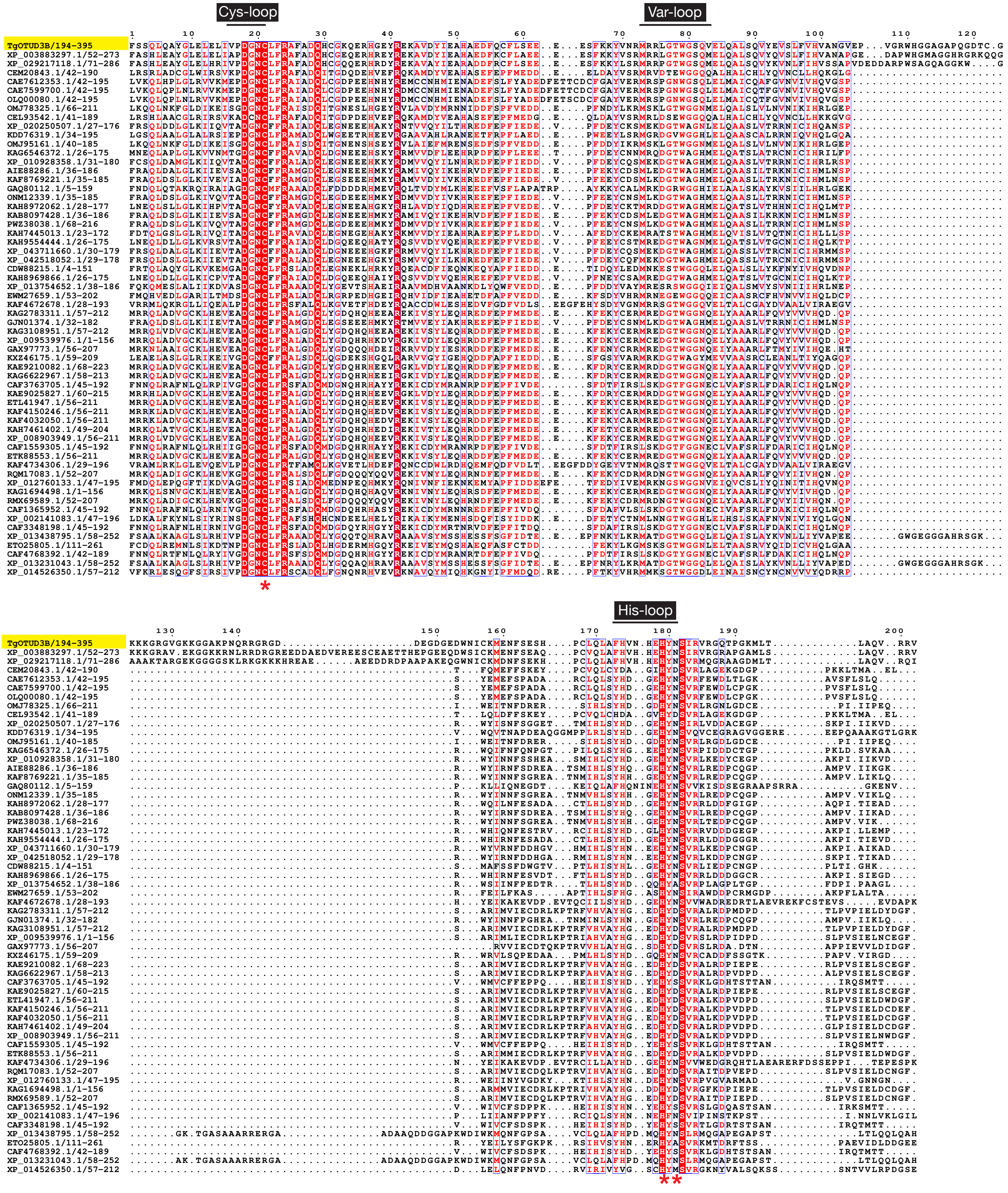

# TgOTUD3C

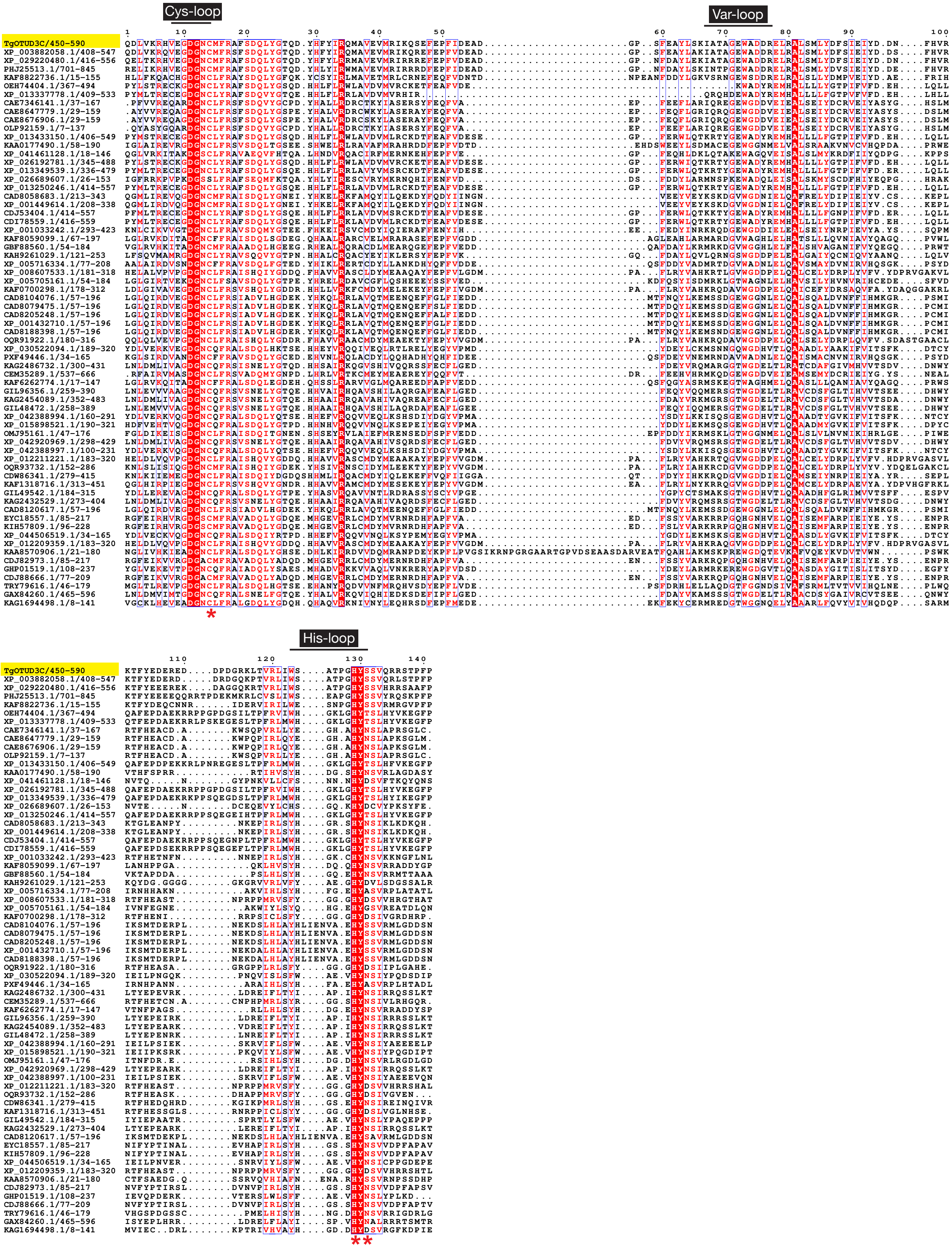

# TgOTUD6A

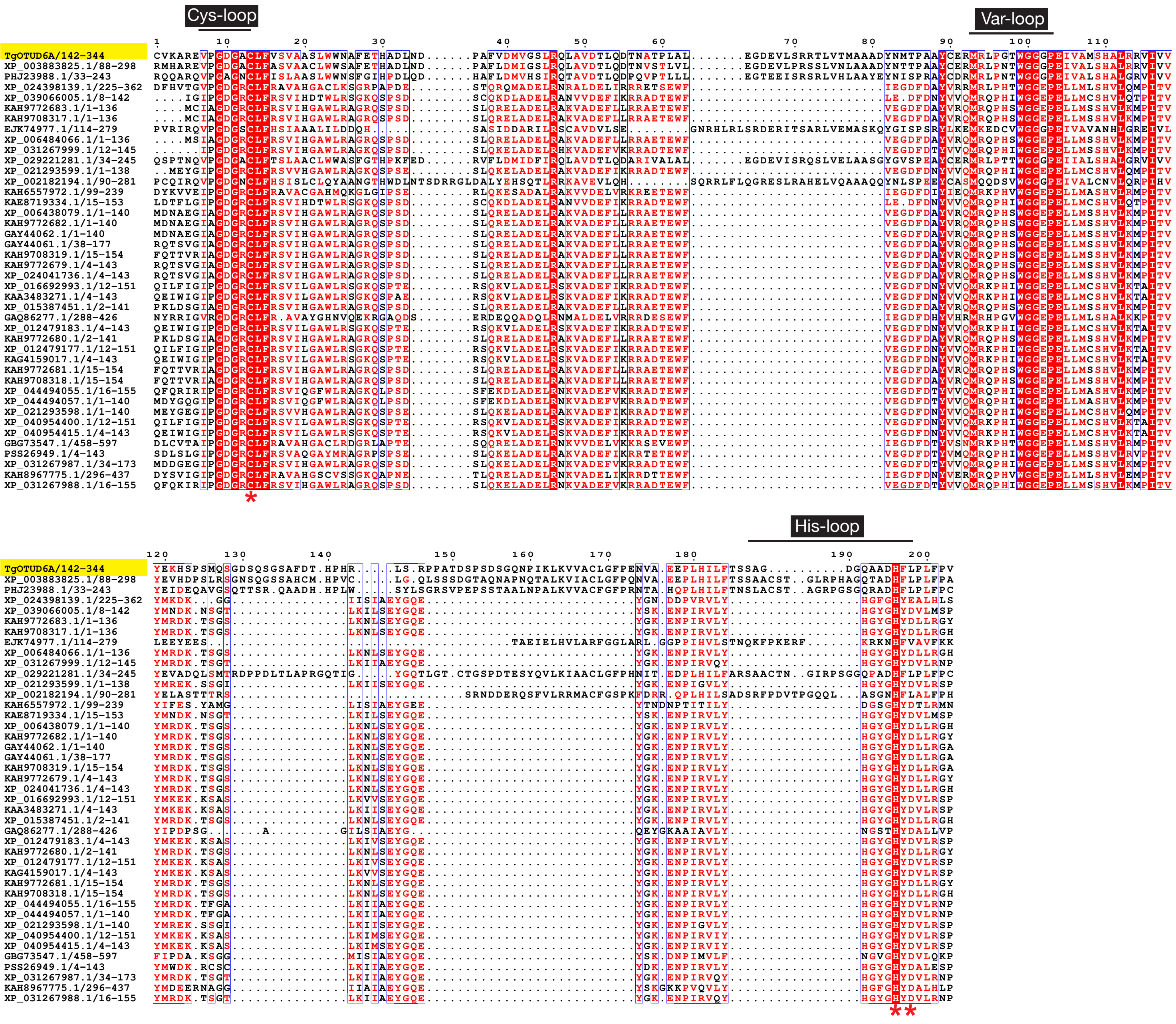

# TgOTUD6B

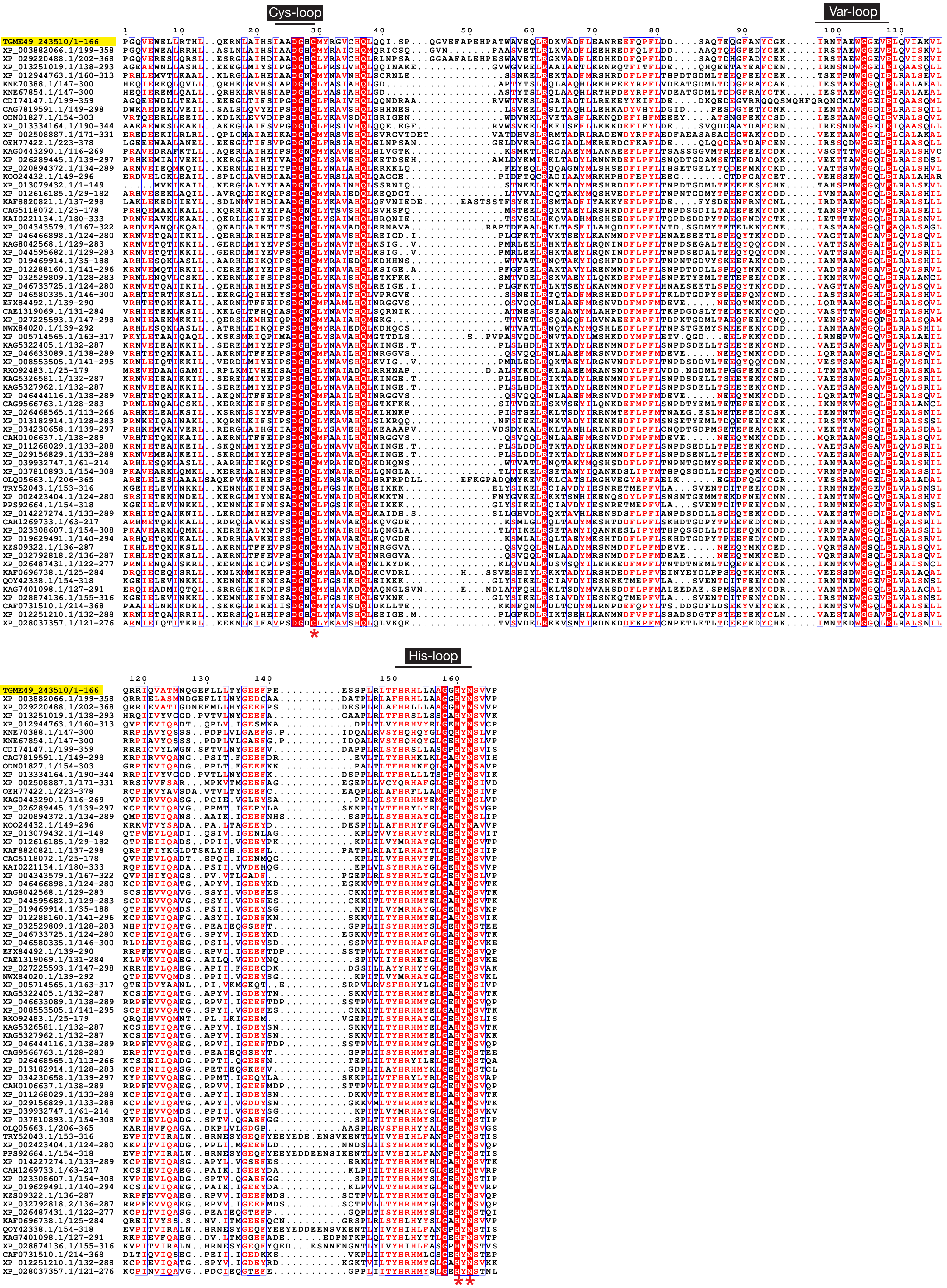

# TgOTUB1

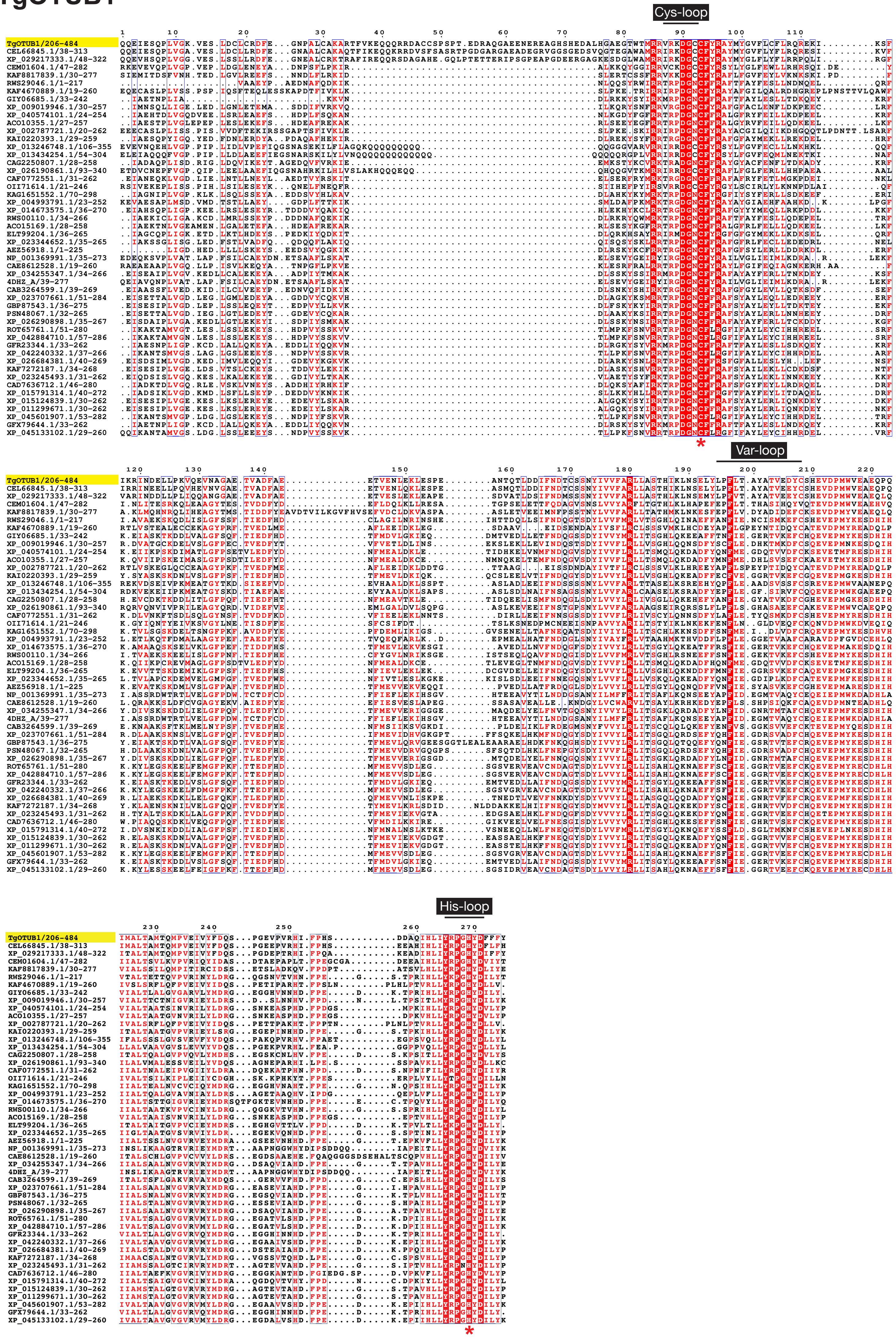

# TgOTU8

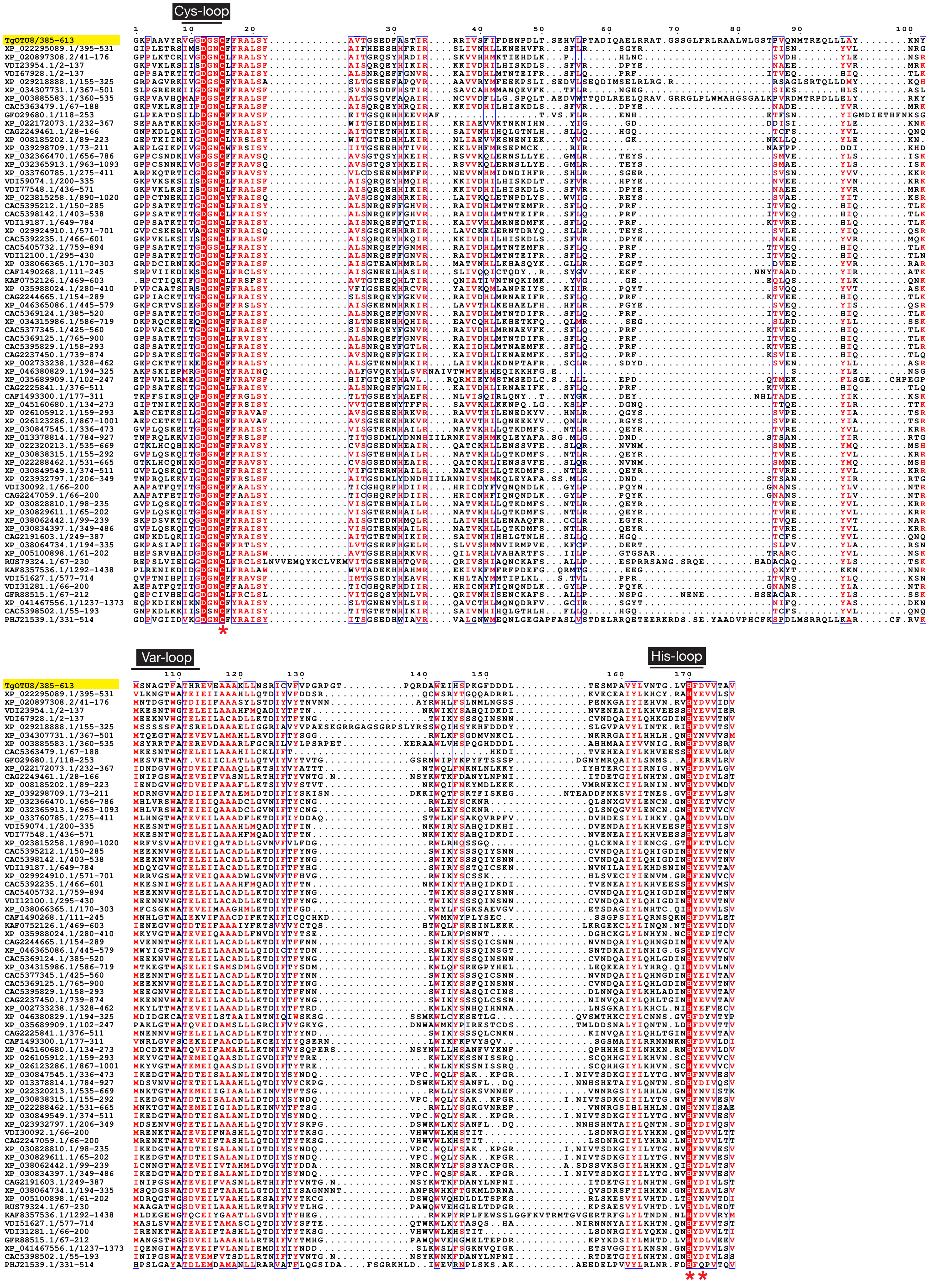

# TgOTU9

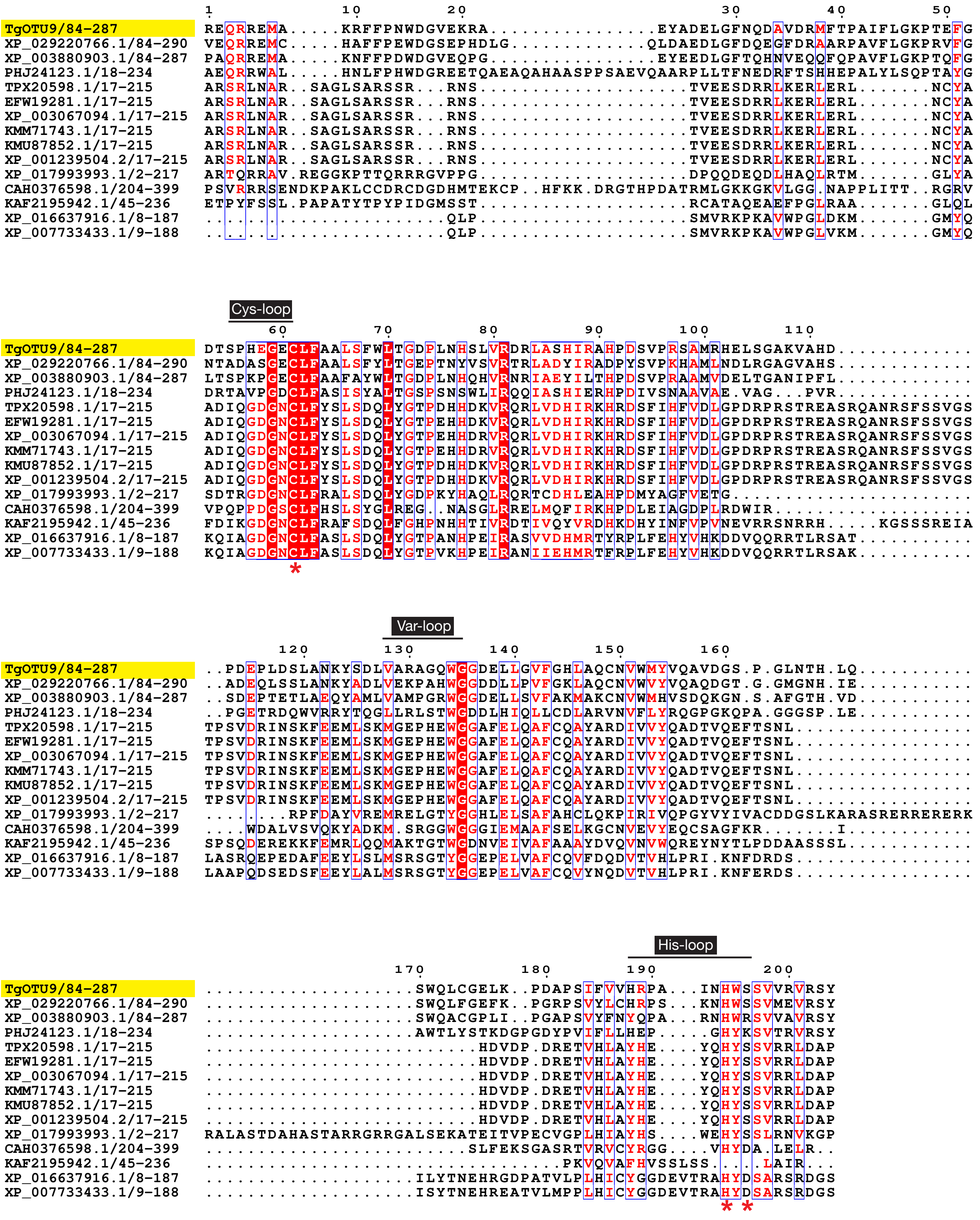

1 10 20 30 40 50
TgOTU9/84-287 REQRREMA....KRFFPNWDGVEKRA...............EYADELGFNQDAVDRMFTPAIFLGKPTEFG
XP_029220766.1/84-290 VEQRREMC....HAFFPEWDGSEPHDLG............QLDAEDLGFDQEGFDRAARPAVFLGKPRVFG
XP_003880903.1/84-287 PAQRREMA....KNFFPDWDGVEQPG..............EYEEDLGFTQHNVEQQFQPAVFLGKPTQFG
PHJ24123.1/18-234 AEQRRWAL....HNLFPHWDGREETQAEAQAHAASPPSAEVQAARPLLTFNEDRFTSHHEPALYLSQPTAYG
TPX20598.1/17-215 ARSRLNAR..SAGLSARSSR...RNS..................TVEESDRRLKERLERL.......NCYA
EFW19281.1/17-215 ARSRLNAR..SAGLSARSSR...RNS..................TVEESDRRLKERLERL.......NCYA
XP_003067094.1/17-215 ARSRLNAR..SAGLSARSSR...RNS..................TVEESDRRLKERLERL.......NCYA
KMM71743.1/17-215 ARSRLNAR..SAGLSARSSR...RNS..................TVEESDRRLKERLERL.......NCYA
KMU87852.1/17-215 ARSRLNAR..SAGLSARSSR...RNS..................TVEESDRRLKERLERL.......NCYA
XP_001239504.2/17-215 ARSRLNAR..SAGLSARSSR...RNS..................TVEESDRRLKERLERL.......NCYA
XP_017993993.1/2-217 ARTQRRAV.REGGKPTTQRRRGVPPG..................DPQQDEQDLHAQLRTM.......GLYA
CAH0376598.1/204-399 PSVRRRSENDKPAKLCCDRCDGDHMTEKCP..HFKK.DRGTHPDATRMLGKKGKVLGG.NAPPLITT..RGRV
KAF2195942.1/45-236 ETPYFSSL.PAPATYTPYPIDGMSST..................RCATAQEAEFPGLRAA.......GLQL
XP_016637916.1/8-187 ......................QLP...................SMVRKPKAVWPGLDKM.......GMYQ
XP_007733433.1/9-188 ......................QLP...................SMVRKPKAVWPGLVKM.......GMYQ

Cys-loop
60 70 80 90 100 110
TgOTU9/84-287 DTSPHEGECLFAALSFWLTGDPLNHSLVRDRLASHIRAHPDSVPRSAMRHELSGAKVAHD............
XP_029220766.1/84-290 NTADASGECLFAALSFYLTGEPTNYVSVRTRLADYIRADPYSVPKHAMLNDLRGAGVAHS............
XP_003880903.1/84-287 LTSPKPGECLFAAFAYWLTGDPLNHQHVRNRIAEYILTHPDSVPRAAMVDELTGANIPFL............
PHJ24123.1/18-234 DRTAVPGDCLFASISYALTGSPSNSWLIRQQIASHIERHPDIVSNAAVAE.VAG...PVR............
TPX20598.1/17-215 ADIQGDGNCLFYSLSDQLYGTPDHHDKVRQRLVDHIRKHRDSFIHFVDLGPDRPRSTREASRQANRSFSSVGS
EFW19281.1/17-215 ADIQGDGNCLFYSLSDQLYGTPEHHDKVRQRLVDHIRKHRDSFIHFVDLGPDRPRSTREASRQANRSFSSVGS
XP_003067094.1/17-215 ADIQGDGNCLFYSLSDQLYGTPEHHDRVRQRLVDHIRKHRDSFIHFVDLGPDRPRSTREASRQANRSFSSVGS
KMM71743.1/17-215 ADIQGDGNCLFYSLSDQLYGTPEHHDRVRQRLVDHIRKHRDSFIHFVDLGPDRPRSTREASRQANRSFSSVGS
KMU87852.1/17-215 ADIQGDGNCLFYSLSDQLYGTPDHHDKVRQRLVDHIRKHRDSFIHFVDLGPDRPRSTREASRQANRSFSSVGS
XP_001239504.2/17-215 ADIQGDGNCLFYSLSDQLYGTPDHHDKVRQRLVDHIRKHRDSFIHFVDLGPDRPRSTREASRQANRSFSSVGS
XP_017993993.1/2-217 SDTRGDGNCLFRALSDQLYGDPKYHAQLRQRTCDHLEAHPDMYAGFVETG....................
CAH0376598.1/204-399 VPQPPDGSCLFHSLSYGLREG..NASGLRRELMQFIRKHPDLEIAGDPLRDWIR..................
KAF2195942.1/45-236 FDIKGDGNCLFRAFSDQLFGHPNHHTIVRDTIVQYVRDHKDHYINFVPVNEVRRSNRRH.....KGSSSREIA
XP_016637916.1/8-187 KQIAGDGNCLFASLSDQLYGTPANHPEIRASVVDHMRTYRPLFEHYVHKDDVQQRRTLRSAT...........
XP_007733433.1/9-188 KQIAGDGNCLFASLSDQLYGTPVKHPEIRANIIEHMRTFRPLFEHYVHKDDVQQRRTLRSAK...........
*

Var-loop
120 130 140 150 160
TgOTU9/84-287 ..PDEPLDSLANKYSDLVARAGQWGGDELLGVFGHLAQCNVWMYVQAVDGS.P.GLNTH.LQ...........
XP_029220766.1/84-290 ..ADEQLSSLANKYADLVEKPAHWGGDDLLPVFGKLAQCNVWVYVQAQDGT.G.GMGNH.IE...........
XP_003880903.1/84-287 ..SDEPTETLAEQYAMLVAMPGRWGGDELLSVFAKMAKCNVWMHVSDQKGN.S.AFGTH.VD...........
PHJ24123.1/18-234 ..PGETRDQWVRRYTQGLLRLSTWGDDLHIQLLCDLARVNVFLYRQGPGKQPA.GGGSP.LE...........
TPX20598.1/17-215 TPSVDRINSKFEEMLSKMGEPHEWGGAFELQAFCQAYARDIVVYQADTVQEFTSNL...............
EFW19281.1/17-215 TPSVDRINSKFEEMLSKMGEPHEWGGAFELQAFCQAYARDIVVYQADTVQEFTSNL...............
XP_003067094.1/17-215 TPSVDRINSKFEEMLSKMGEPHEWGGAFELQAFCQAYARDIVVYQADTVQEFTSNL...............
KMM71743.1/17-215 TPSVDRINSKFEEMLSKMGEPHEWGGAFELQAFCQAYARDIVVYQADTVQEFTSNL...............
KMU87852.1/17-215 TPSVDRINSKFEEMLSKMGEPHEWGGAFELQAFCQAYARDIVVYQADTVQEFTSNL...............
XP_001239504.2/17-215 TPSVDRINSKFEEMLSKMGEPHEWGGAFELQAFCQAYARDIVVYQADTVQEFTSNL...............
XP_017993993.1/2-217 ........RPFDAYVREMRELGTYGGHLELSAFAHCLQKPIRIVQPGYVYIVACDDGSLKARASRERRERERK
CAH0376598.1/204-399 ....WDALVSVQKYADKM.SRGGWGGIEMAAFSELKGCNVEVYEQCSAGFKR.......I.............
KAF2195942.1/45-236 SPSQDEREKKFEMRLQQMAKTGTWGDNVEIVAFAAAYDVQVNVWQREYNYTLPDDAASSSL............
XP_016637916.1/8-187 LASRQEPEDAFEEYLSLMSRSGTYGGEPELVAFCQVFDQDVTVHLPRI.KNFDRDS...............
XP_007733433.1/9-188 LAAPQDSEDSFEEYLALMSRSGTYGGEPELVAFCQVYNQDVTVHLPRI.KNFERDS...............

His-loop
170 180 190 200
TgOTU9/84-287 .....................SWQLCGELK..PDAPSIFVVHRPA...INHWSSVVRVRSY
XP_029220766.1/84-290 .....................SWQLFGEFK..PGRPSVYLCHRPS...KNHWSSVMEVRSY
XP_003880903.1/84-287 .....................SWQACGPLI..PGAPSVYFNYQPA...RNHWRSVVAVRSY
PHJ24123.1/18-234 .....................AWTLYSTKDGPGDYPVIFLLHEP....GHYKSVTRVRSY
TPX20598.1/17-215 ...........................HDVDP.DRETVHLAYHE....YQHYSSVRRLDAP
EFW19281.1/17-215 ...........................HDVDP.DRETVHLAYHE....YQHYSSVRRLDAP
XP_003067094.1/17-215 ...........................HDVDP.DRETVHLAYHE....YQHYSSVRRLDAP
KMM71743.1/17-215 ...........................HDVDP.DRETVHLAYHE....YQHYSSVRRLDAP
KMU87852.1/17-215 ...........................HDVDP.DRETVHLAYHE....YQHYSSVRRLDAP
XP_001239504.2/17-215 ...........................HDVDP.DRETVHLAYHE....YQHYSSVRRLDAP
XP_017993993.1/2-217 RALASTDAHASTARRGRRGALSEKATEITVPECVGPLHIAYHS....WEHYSSLRNVKGP
CAH0376598.1/204-399 ..........................SLFEKSGASRTVRVCYRGG....VHYDA.LELR..
KAF2195942.1/45-236 ..................................PKVQVAFHVSSLSS.......LAIR...
XP_016637916.1/8-187 .....................ILYTNEHRGDPATVLPLHICYGGDEVTRAHYDSARSRDGS
XP_007733433.1/9-188 .....................ISYTNEHREATVLMPPLHICYGGDEVTRAHYDSARSRDGS
* *

# TgOTU10

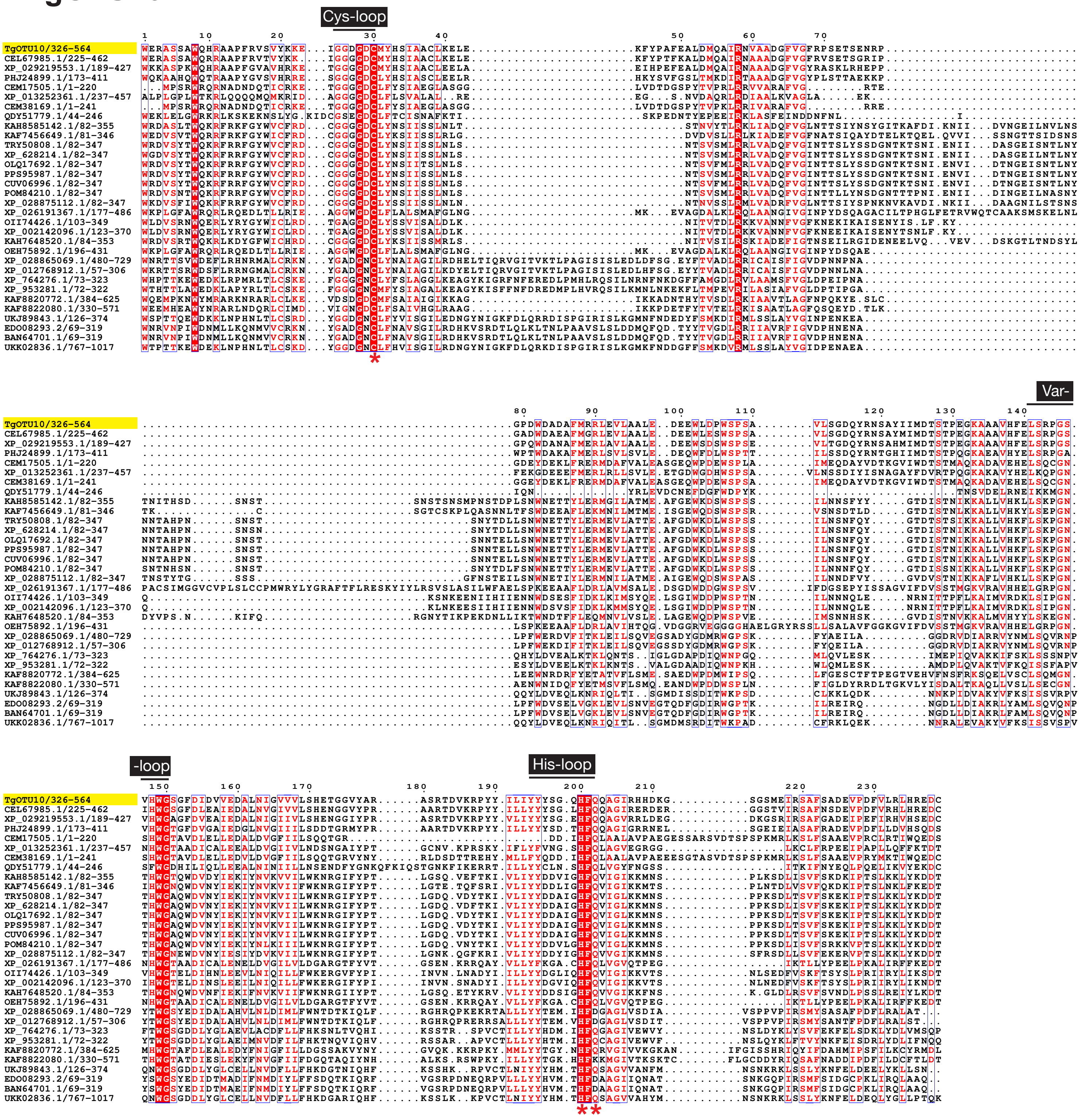

Cys-loop
Var-
-loop
His-loop
TgOTU10/326-564
CEL67985.1/225-462
XP_029219553.1/189-427
PHJ24899.1/173-411
CEM17505.1/1-220
XP_013252361.1/237-457
CEM38169.1/1-241
QDY51779.1/44-246
KAH8585142.1/82-355
KAF7456649.1/81-346
TRY50808.1/82-347
XP_628214.1/82-347
OLQ17692.1/82-347
PPS95987.1/82-347
CUV06996.1/82-347
POM84210.1/82-347
XP_028875112.1/82-347
XP_026191367.1/177-486
OII74426.1/103-349
XP_002142096.1/123-370
KAH7648520.1/84-353
OEH75892.1/196-431
XP_028865069.1/480-729
XP_012768912.1/57-306
XP_764276.1/73-323
XP_953281.1/72-322
KAF8820772.1/384-625
KAF8822080.1/330-571
UKJ89843.1/126-374
EDO08293.2/69-319
BAN64701.1/69-319
UKK02836.1/767-1017
